# An Improved Genetic Algorithm for Solving the Multi-AGV Flexible Job Shop Scheduling Problem

**DOI:** 10.3390/s23083815

**Published:** 2023-04-07

**Authors:** Leilei Meng, Weiyao Cheng, Biao Zhang, Wenqiang Zou, Weikang Fang, Peng Duan

**Affiliations:** 1 School of Computer Science, Liaocheng University, Liaocheng 252000, China; 2 School of Mechanical Science and Engineering, Huazhong University of Science and Technology, Wuhan 430074, China

**Keywords:** flexible job shop scheduling problem, automatic guided vehicle, genetic algorithm

## Abstract

In real manufacturing environments, the number of automatic guided vehicles (AGV) is limited. Therefore, the scheduling problem that considers a limited number of AGVs is much nearer to real production and very important. In this paper, we studied the flexible job shop scheduling problem with a limited number of AGVs (FJSP-AGV) and propose an improved genetic algorithm (IGA) to minimize makespan. Compared with the classical genetic algorithm, a population diversity check method was specifically designed in IGA. To evaluate the effectiveness and efficiency of IGA, it was compared with the state-of-the-art algorithms for solving five sets of benchmark instances. Experimental results show that the proposed IGA outperforms the state-of-the-art algorithms. More importantly, the current best solutions of 34 benchmark instances of four data sets were updated.

## 1. Introduction

The flexible job shop scheduling problem (FJSP) widely exists in the modern manufacturing workshop and is becoming more and more important. For FJSP, an operation can selected to be machined by a set of machines instead of one. Therefore, two sub-problems, namely machine selection and operations sequencing must be determined. Moreover, FJSP has proved to be an NP-hard problem [1,2]. In real production, there is a certain distance between different machines. The jobs must be transformed by automatic guided vehicles (AGVs) from machines to machines. Due to the high price of AGVs and the workshop layout, the number of AGVs is limited. The FJSP with a limited number of AGVs (FJSP-AGV) is much nearer to real production than FJSP and very important. Compared with FJSP, FJSP-AGV should solve three sub-problems, namely the machine selection sub-problem, the AGV selection sub-problem, and the operations sequencing sub-problem. Therefore, FJSP-AGV is a much more difficult NP-hard problem than FJSP [3].

The genetic algorithm (GA) is inspired by the process of natural selection and has been widely implemented to solve shop scheduling problems [4,5]. Moreover, GA shows good effectiveness for solving FJSP [6,7] and therefore, can be used for solving FJSP-AGV. With regard to the classical GA, the diversity of the population decreases with the algorithm iteration, and some individuals can become extremely similar, even identical, causing stagnation of population evolution. In order to overcome this problem, an improved GA (IGA) with a population diversity check method was specifically designed. Comparison experiments of benchmark instances were conducted to evaluate the effectiveness and efficiency of IGA. What is more, the proposed IGA can update the current best solutions of 34 benchmark instances.

The rest of this paper is presented as follows: Section 2 introduces the literature review of FJSP-AGV; Section 3 describes the FJSP-AGV; Section 4 presents the IGA from several aspects in detail; Section 5 displays the experimental results; Section 6 contains the conclusions and proposed future work.

## 2. Literature Review

For solving the scheduling problem, there are mainly two types of algorithms, namely the exact algorithm and the approximation algorithm. With regard to the exact algorithm, mixed integer linear programming (MILP) is commonly employed [8,9,10]. As to approximation methods, meta-heuristic algorithms are mostly used [11,12,13,14,15,16]. With regard to FJSP, GA is used in the main and has proved to be effective [6].

### 2.1. Literature Review of GA for Solving FJSP 

Job shop scheduling problem (JSP) is the basis of FJSP, and does not consider the flexibility of machine selection. With minimizing makespan of JSP, Zhang et al. [17] proposed an effective hybrid GA, Xie et al. [18] developed a new improved GA that combines GA and tabu search (TS), and Goncalves et al. designed a random-key based GA. With regard to FJSP with minimizing makespan, Pezzella et al. [19] designed a GA with different rules for generating individuals, Zhang et al. proposed a combined GA that takes variable neighborhood search into consideration, Gutiérrez et al. designed a hybrid GA that combines GA and repair heuristics, and Fan et al. [20] developed an improved genetic algorithm, in which problem-specific encoding and decoding strategies are designed. 

### 2.2. Literature Review of FJSP-AGV

JSP with a limited number of AGV is named as JSP-AGV. With regard to JSP-AGV, the existing works have been focused on minimizing makespan. Bilge and Ulusoy [21] designed an iterative algorithm and a set of benchmark instances. Erol et al. [22] developed a multi-agent-based algorithm, which includes four agents, namely manager agent, staff agent, AGV agent, machine agent, and AGV-machine resource agents. Deroussi et al. [23] designed a novel neighboring method, which includes three intelligent algorithms, namely iterated local search, simulated annealing, and their hybridization. Kumar et al. [24] developed a novel differential evolution algorithm, whose encoding only considers the operations sequencing sub-problem. Moreover, the machine selection sub-problem and the AGV selection sub-problem are determined in the decoding with specific heuristics. Zheng et al. [25] designed a tabu search algorithm and first presented a mixed integer linear programming (MILP) model to obtain optimal solutions. Fontes and Homayouni [26] proposed an improved MILP by considering more constraints for minimizing makespan of JSP-AGV. Abdelmaguid et al. [27] proposed a hybrid GA/heuristic approach, while the heuristic is to determine the AGV selection in the decoding scheme. Lacomme et al. [28] designed a disjunctive graph-based framework for modeling JSP-AGV and an improved memetic algorithm. Ham [29] first developed a constraint programming model and a new set of benchmark instances. 

In order to simultaneously optimize makespan, mean flow time, and mean tardiness of JSP-AGV, an improved multi-objective GA was designed [30], which determines the AGV selection sub-problem in the decoding. With regard to JSP-AGV simultaneously making makespan, AGV travel time, and minimized penalty cost, a multi-objective GA was designed, which used the fuzzy expert system to adjust crossover operators [31]. With consideration of the battery charge of AGV, three intelligent algorithms, namely GA, particle swarm optimization (PSO), and hybrid GA-PSO were developed to simultaneously make makespan with the number of AGV being minimized [32]. 

With only one AGV in a flexible manufacturing system, Caumond et al. [33] proposed a MILP for scheduling problems. Moreover, the maximum number of jobs, the limited input/output buffer capacities, and the no-move-ahead trips were taken into consideration simultaneously. With regard to FJSP-AGV on minimizing makespan, Ham [29] extended the constraint programming model of JSP-AGV and proved the optimality of ten benchmark instances [34]. Homayouni and Fontes [3] proposed the first MILP model for solving small-sized instances to optimality and a local search-based heuristic for solving small to large-sized instances. Chaudhry et al. [35] presented a Microsoft Excel spreadsheet-based solution and GA, and Homayouni et al. [11] proposed a multi-start biased random key genetic algorithm (BRKGA). In BRKGA, the encoding only considers the operations sequencing sub-problem, and the machine selection and AGV selection sub-problems are determined by several different greedy heuristics. Zhang et al. [36] designed a hybrid algorithm GATS that combines GA and tabu search algorithm (TS) for minimizing makespan and of FJSP-AGV with bounded processing times. In GATS, the GA decides the machine-AGV selections of all operations and TS optimizes the operations sequencing. In order to fast and accurately estimate the makespan of FJSP-AGV, Cheng et al. [37] designed an adaptive ensemble model of back propagation neural networks. Yan et al. [38] first studied the FJSP-AGV in a digital twin workshop and developed a three-layer -encoding based GA. Moreover, in order to implement the optimized schedules to a digital twin system, an entity-JavaScript Object Notation method was designed. As we know, Li et al. [12] first studied dynamic FJSP-AGV with simultaneously minimizing makespan and total energy consumption and developed a hybrid deep Q network (HDQN)-based dynamic scheduling method.

## 3. Problem Description

The FJSP-AGV problem studied in this paper is the same as with the existing research [3]. FJSP-AGV includes a set of jobs, machines, and AGVs. For each job, it includes several operations and must abide by its predefined route. For each operation, it can select being machined on different machines and transported by different AGVs. When the two adjacent operations of a job are not assigned to the same machine, the job must be transported by one AGV. All the AGVs are identical, and each of them can transport at most one job at the same time. Moreover, the objective of this work was to determine the machine selection sub-problem, the AGV selection sub-problem, and the operations sequencing sub-problem so as to make the makespan minimized. 

**Figure 1:**
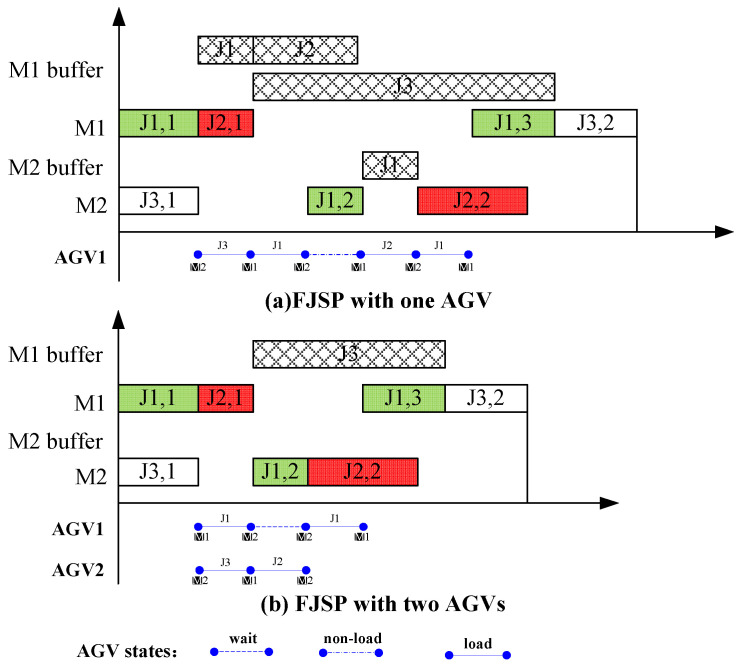
An example of the FJSP-AGV.

To intuitively show the FJSP-AGV, an example is given in Figure 1. Specifically, Figure 1a shows the FJSP with only one AGV and Figure 1b shows the FJSP with two AGVs. As we can see from Figure 1a, the AGV1 first transfers Job 3 from Machine 2 to Machine 1, then transfers Job 1 from Machine 1 to Machine 2, then returns to Machine 1 transferring no job, then transfers Job 2 from Machine 1 to Machine 2, and finally transfers Job 1 from Machine 2 to Machine 1. As can be seen from Figure 1b, AGV1 first transfers Job 1 from Machine 1 to Machine 2, then waits for some time, and finally transfers Job 1 from Machine 2 to Machine 1. AGV2 first transfers Job 3 from Machine 2 to Machine 1, and then transfers Job 2 from Machine 1 to Machine 2.

## 4. The Improved Genetic Algorithm

### 4.1. Initialization

Initialization of the GA includes the population and the parameters. With regard to the initial population, all the individuals are generated randomly according to the following encoding methods. The parameters include the population size *N*, the cross probability *Pc*, the mutation probability *Pm,* and the stopping criteria.

### 4.2. Encoding Scheme

Encoding explains how to represent a real solution. Encoding of the individual is very important in GA. In this paper, the encoding method that is usually used for FJSP is also used [6]. The encoding only considers two strings, namely operation sequence (OS) string and machine selection (MS) string, and not considering the AGV string. The operation sequencing subproblem is determined by the OS string, and the machine selection subproblem is determined by the MS string. With regard to the AGV selection subproblem, this is determined in the decoding scheme with specifically designed rules.

**Figure 2:**
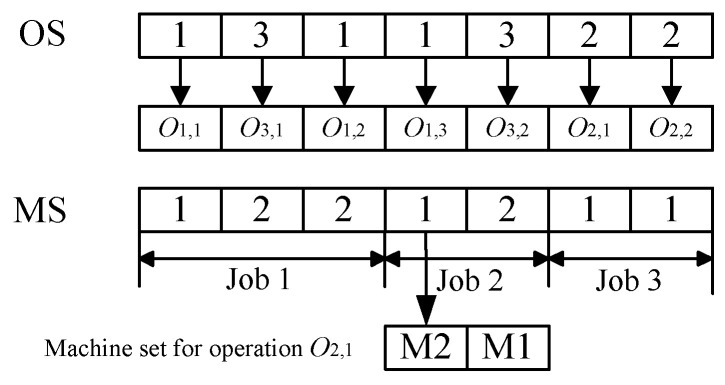
The encoding chromosome.

The OS string defines all operations of a job with the same symbol and then interprets them according to the sequence of their appearance, the length of which is equal to the total number of operations. The genes of the MS string describe the selected machines of the corresponding operations, whose length is also equal to the total number of operations. It is important to note that each element of MS does not represent the actual machine number but the index in the matrix of the alternative machine set. Figure 2 shows an example illustrating the encoding method. With regard to the MS string, for example, the machine index of operation $O_{2,1}$ is 1, and corresponds to the real Machine 2.

### 4.3. Decoding Scheme

Decoding is to transform a chromosome to a real schedule. The heuristic for determining the AGV selection is designed as follows:

(1) With regard to the first operation of a job, of the AGVs that arrive at the machine $m_{i,1}$ the earliest is selected.
  (1) $$v*=\underset{v\in V}{\arg\min}\{t_{v}+T_{l_{v}}^{LU}+T_{LU}^{m_{i,1}}\}$$
  where, $t_{v}$ and $l_{v}$ denote the times that AGV v becomes available to transport its next operation and its location respectively when it finishes its previous operation. Specifically, the initial $t_{v}$ is 0, and the initial location of AGV v is LU. $T_{l_{1}}^{l_{2}}$ denotes the transportation time between locations $l_{1}$ and $l_{2}$. Obviously, if $l_{1}=l_{2}$, then $T_{l_{1}}^{l_{2}}=0$. $m_{i,j}$ denotes the selected machine for processing operation $O_{i,j}$.
(2) With regard to other operations of a job, of the AGVs that arrive at the machine $m_{i,j}$ the earliest is selected.
  (2) $$v*=\underset{v\in V}{\arg\min}\{t_{v}+T_{l_{v}}^{m_{i,j-1}}+T_{m_{i,j-1}}^{m_{i,j}}\}$$

Specifically, when two or more AGVs are available to transport an operation at the same time, the first AGV is selected.

The decoding starts from the first operation to the last operation, and the related times are updated from the following Equations (3)–(5).

(3) $$d_{v}=\max\{t_{v}+T_{l_{v}}^{m_{i,j-1}},c_{i,j-1}\}$$

(4) $$t_{v}=d_{v}+T_{m_{i,j-1}}^{m_{i,j}}$$

(5) $$c_{i,j}=\max\{c_{m_{i,j}},t_{v}\}+p_{{}_{i,j}}^{m_{i,j}}$$

where, $d_{v}$ denotes the time that AGV v leaves machine $m_{i,j-1}$. $c_{m_{i,j}}$ represents the time that machine $m_{i,j}$ finishes the current operation immediately before operation $O_{i,j}$. $c_{i,j}$ indicates the finishing time of operation $O_{i,j}$.

### 4.4. Selection Operator

In IGA, the role of selection operator is to select the individuals according to the fitness (makespan in this paper). For the purpose of this paper, we adopt two selection operators namely the elitist selection and the binary tournament selection [6]. The elitist selection aims to preserve the individual with the best fitness to the offspring. Specifically, the first two best individuals are directly preserved from the parent population to the offspring population. Except for the best two individuals, the other *N*-2 individuals in the offspring population are generated by using binary tournament selection, which works by selecting two individuals from the population and selecting the one with better fitness. For example, parent P1\P2 is generated by selecting the better one of two randomly selected individuals in the parent population. Then, offspring O1\O2 is generated from the parent P1\P2 by conducting crossover and mutation operators.

### 4.5. Crossover Operator

**Figure 3:**
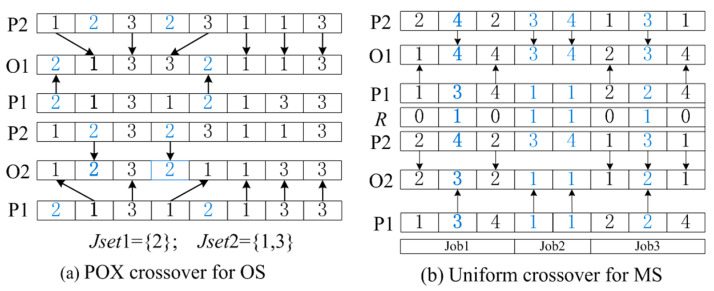
Example of POX and UC for OS and MS respectively. (**a**), each arrow denotes one OS, and the jobs of different subsets are shown in different color. (**b**), each arrow denotes one MS, and the selected and non-selected machines are shown in different color.

In this work, the two mostly used crossovers for OS string and MS string are adopted, namely precedence operation crossover (POX) and uniform crossover (UC). Specifically, POX is for OS string, and its steps are given as follows: First, all the jobs are randomly divided into two subsets, namely Jset1 and Jset2. Then, the jobs of parent P1\P2 that are the parts of Jset1\Jset2 are preserved to offspring O1\O2 keeping their positions unchanged. Finally, the other jobs of parent P2\P1 that are not parts of Jset1\Jset2 are copied to offspring O1\O2 keeping their positions unchanged. To intuitively show the POX, a small example is given in Figure 3a.

With regard to UC, this is used for the MS string, and its steps are as follows: First, a certain number of binary numbers is randomly generated. Then, the offspring O1\O2 is obtained by swapping the machine selections of parent P1\P2, whose binary numbers equal 1. Moreover, a small example is given in Figure 3b to intuitively show the UC.

### 4.6. Mutation Operator

In this paper, swap mutation and one-point-reassign mutation were adopted for the OS string and MS string respectively [6]. Swap mutation works by randomly selecting two different positions and exchanging their elements. With regard to one-point-reassign mutation, one position is randomly selected and then its value is changed to another eligible machine. These two mutation operators are selected randomly with the same 50% probability.

### 4.7. Population Diversity Check

With regard to the classical GA, the diversity of the population decreases with the algorithm iteration, and some individuals may become extremely similar even identical, causing stagnation of population evolution. In order to make up the gap, at regular intervals, the population is checked and the similar individuals are randomly generated. Specifically, if two individuals have the same makespan and the similarity of their MS strings is no less than 80%, one of them must be regenerated. If in each generation, the population diversity is checked, it will be very time-consuming. Therefore, we check the population diversity at each *Nt* generation.

### 4.8. The Steps of the Proposed IGA

The steps of the proposed IGA are as follows:

- **Step 1**: Initialization: Initialize the parameters and the initial population of IGA, and t=1.
- **Step 2**: Evaluation: Evaluate all the individuals by the fitness of total energy consumption.
- **Step 3**: Genetic evolutions: Execute the genetic operations, namely selection in Section 4.4, crossover in Section 4.5, and mutation in Section 4.6.
- **Step 4**: Population diversity check: Check the population diversity *Nt* generations according to the methods in Section 4.7.
- **Step 5**: t=t+1.
- **Step 6**: Termination: Has the stopping criteria been reached? If the stopping criteria is met, go to Step 7; otherwise, go to Step 2.
- **Step 7**: Output the best solution.

**Figure 4:**
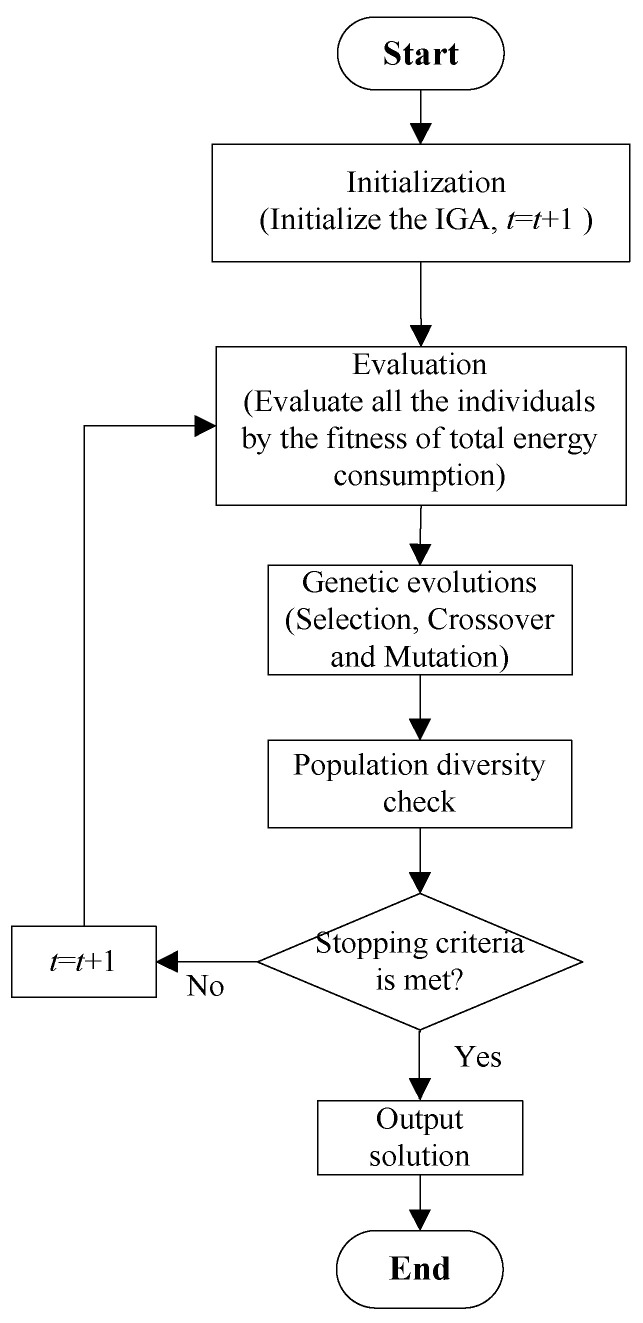
The flow chart of IGA.

Figure 4 shows the flow chart of the proposed IGA.

### 4.9. Computational Complexity Analysis

With regard to IGA computational complexity, this is determined by each step of the IGA. In detail, the complexity of initialization is $O(N_{oper}\times N)$. The complexity of binary tournament selection is O(N). The complexity of POX and UC are $O(N_{oper})$. The complexity of swap mutation and one-point-reassign mutation are O(1). The complexity of the population diversity check is $O(N_{oper}\times N^{2})$. Therefore, the final computational complexity of IGA is mainly determined by $N_{oper}$ and N.

## 5. Experimental Results

The IGA was coded in C++ and run on a computer with the Win 11 system, Intel(R) Core(TM) i7-10700 CPU @ 2.90 GHz and 24 GB of RAM memory. Experimental tests were conducted based on five sets of benchmark data. For each instance, the test was repeated 20 times. The stopping criterion was set as maximum CPU time of $2N_{oper}$ seconds, and $N_{oper}$ is the number of the total operations of all the jobs in the instance. For IGA, the parameters of *N*, *Nt*, *Pc,* and *Pm* are tried to be selected from {200, 300, 400}, {100, 300, 300}, {0.7, 0.8, 0.9} and {0.05, 0.1, 0.2} respectively. In a lot of experiments, *N*, *Nt*, *Pc,* and *Pm* were set to 200, 200, 0.8, and 0.1 respectively. 

To evaluate the effectiveness of the population diversity check method, the IGA was compared with the classical GA without considering the population diversity check method. The comparison results of MFJST01-10 and MKT01-10 [3] are shown in Table 1. Specifically, we set the relative percentage increase (RPI) as the comparison indicator, as is shown in Table 1:

(6) $$RPI=\frac{MC-MC_{best}}{MC_{best}}\times 100$$

where, MC represents the result of a test instance that is obtained by a specific algorithm by repeating several times, and $MC_{best}$ denotes the best MC of all comparison algorithms.

In Table 1, the “Best”, “Mean” and “Worst” represent the best value, the mean value, and the worst value of repeats of 20 times. The “Mean RPI” represents the mean value of PRI for all the instances. As can be seen from Table 1, IGA outperforms GA in terms of the best value, the mean value, and the worst value. With regard to MFJST01, GA can obtain the same best solution 485 as IGA. GA cannot obtain the best solution 485 of 20 times, and its worst solution is 517. Compared with GA, the IGA can obtain the best solution 485 of 20 times. With other instances, IGA and GA cannot obtain the same solution of 20 times. This is because the solution spaces of the instances increase greatly as the problem size is increased. 

To prove the effectiveness and efficiency of the proposed IGA, it is compared with state-of-the-art algorithms, namely, the MILP model [3], CP2 [29], LAHC [3], BRKGA [11], GATS [36], and PGA [35]. The comparison results of the five sets of benchmark data sets are shown in Table 2, Table 3, Table 4, Table 5, Table 6 and Table 7. Moreover, in Table 2, Table 3, Table 4, Table 5, Table 6 and Table 7, the solutions in bold are the best among all the algorithms, the solutions with “*” are the improved ones by our IGA, and the solutions with “-” are worse than the best-known solutions. In the Appendix A, we have given detailed information of some improved solutions.

### 5.1. Comparison Results of Data Set 1

The comparison results of data set 1 are shown in Table 2. Specifically, CP2 has proved the optimal solutions of all the ten instances. As shown in Table 2, our proposed IGA can obtain all the optimal solutions for the 10 instances. The existing meta-heuristic algorithms LAHC, BRKGA, and GATS can only obtain 9, 4, and 2 optimal solutions. Obviously, except for the exact algorithm CP2, our IGA is the only meta-heuristic algorithm that can solve all the instances to optimality.

### 5.2. Comparison Results of Data Set 2

The comparison results of data set 2 are shown in Table 3 and Table 4. Specifically, Table 3 shows the results of data set 2 with *t*/*p* > 0.25, and Table 4 shows the results of data set 2 with *t*/*p* ≤ 0.25. In Table 3 and Table 4, the obtained solutions of MILP model are optimal. As can be seen from Table 3, our proposed IGA outperforms all the existing algorithms and updates the current best solutions of four instances, namely, EX81, EX72, EX82, and EX83, and their best solutions are improved from 94, 62, 82, 85 and 87 to 91, 61, 80 and 84 respectively. Except the improved four instances, our IGA can obtain the best solutions for all the other 24 instances, and the existing meta-heuristic algorithms LAHC, BRKGA, and PGA can only obtain 22, 9, and 15 best solutions.

As presented in Table 4, our proposed IGA outperforms all the existing algorithms and improves the current best solutions of three instances, namely, EX730, EX741, and EX840. Specifically, our proposed IGA improves the upper bounds of EX730, EX741, and EX840 from 100, 150, and 144 to 99, 149, and 143 respectively. Except the improved three instances, our IGA can obtain the best solutions for all the other 26 instances. The existing meta-heuristic algorithms LAHC, BRKGA, and PGA can only obtain 18, 14, and 14 best solutions.

### 5.3. Comparison Results of Data Set 3

The comparison results of data set 3 are shown in Table 5, and the obtained solutions of the MILP model are optimal. As shown in Table 5, our proposed IGA outperforms all the existing algorithms and updates the current best solutions of MFJST09 and MFJST10. For MFJST09 and MFJST10, our proposed IGA improves their best solutions from 1120 and 1238 to 1117 and 1228 respectively. For all the other eight instances, our IGA can obtain all their best solutions, and the existing meta-heuristic algorithms LAHC and PGA can only obtain 8 and 4 best solutions.

### 5.4. Comparison Results of Data Set 4

The comparison results of data set 4 are shown in Table 6. From Table 6, we can see that our proposed IGA improves the current best solutions of 9 out of 10 instances. With regard to MKT05, the solution 244 of our proposed IGA is worse than the best solution 225. With regard to MKT01-04 and 06-10, our proposed IGA improved their best solutions from 187, 148, 371, 312, 389.5, 291, 846, 794, and 712.5 to 177, 126, 342, 295, 321.5, 267, 780.5, 715, and 645 respectively.

### 5.5. Comparison Results of Data Set 5

The comparison results of 21 instances of data set 5 are shown in Table 7. As presented in Table 7, our proposed IGA updates the current best solutions of 16 out of 21 instances. For instances mt10cct, mt10xxt, and mt10xyzt, the IGA can obtain the same best solution as existing algorithms. For instances setb4cct and setb4xt, the solutions of IGA are better than these of LAHC (H = 100) and a little worse than LAHC (H = 1000). The reason can be attributed to the decoding heuristic of determining the AGV selection. Not considering AGV selection in the encoding scheme, the solution space of encoding–decoding is limited to some extent. For the instances setb4cct and setb4xt, the best\optimal solutions may not be in the solution space.

## 6. Conclusions and Future Works

This paper studied FJSP-AGV and proposed an IGA to minimize makespan. The IGA was designed specifically from the encoding method, the decoding method, the initiation method of the population, the evolution operators, and the population diversity check method. In order to prove the effectiveness and efficiency of IGA, it was compared with the state-of-the-art algorithms for solving five sets of benchmark instances. Experimental results show that the proposed IGA outperforms the existing algorithms and updates the current best solutions of 34 benchmark instances. Specifically, the proposed IGA updates the current best solutions of four instances of data set 2 with *t*/*p* > 0.25, three instances of data set 2 with *t*/*p* ≤ 0.25, two instances of data set 3, nine instances of data set 4 and 16 instances of data set 5.

In future research, the essential characteristics (e.g., different encoding and decoding schemes) of FJSP-AGV with minimizing makespan will be mined by analyzing the optimal solutions obtained by MILP models. Moreover, more objectives, such as energy-efficiency and cost objectives will be considered.

## Figures and Tables

**Table 1:** Comparison results of IGA and GA.

Instances	Best		Mean		Worst	
	GA	IGA	GA	IGA	GA	IGA
MFJST01	**485**	**485**	498.1	**485.0**	517	**485**
MFJST02	476	**463**	487	**472.7**	509	**478**
MFJST03	491	**482**	510.9	**491.8**	548	**499**
MFJST04	594	**576**	617.5	**593.5**	646	**633**
MFJST05	558	**532**	612.3	**567.6**	644	**604**
MFJST06	660	**652**	708.8	**675.0**	760	**698**
MFJST07	912	**898**	954.5	**928.6**	970	**960**
MFJST08	926	**900**	975.7	**940.6**	1041	**1010**
MFJST09	1147	**1117**	1201.2	**1155.3**	1274	**1195**
MFJST10	1310	**1228**	1346.9	**1311.1**	1393	**1347**
Mean RPI	2.8	**0.0**	4.0	**0.0**	5.5	**0.0**

**Table 2:** Comparison results for the instances in data set 1.

Instances	MILP [3]	CP2 [29]	LAHC (H = 1000) [3]	BRKGA [11]	GATS [36]	IGA
FJSPT1	**134**	**134**	**134**	138	144	**134**
FJSPT2	**114**	**114**	**114**	118	**118**	**114**
FJSPT3	**120**	**120**	**120**	**120**	124	**120**
FJSPT4	**114**	**114**	**114**	120	124	**114**
FJSPT5	**94**	**94**	**94**	96	**94**	**94**
FJSPT6	**138**	**138**	**138**	**138**	144	**138**
FJSPT7	110	**108**	112	112	124	**108**
FJSPT8	**178**	**178**	**178**	**178**	180	**178**
FJSPT9	**144**	**144**	**144**	**144**	150	**144**
FJSPT10	**174**	**174**	**174**	174	178	**174**

**Table 3:** Comparison results for the instances in data set 2 with *t*/*p* > 0.25.

Instances	MILP [3]	LAHC (H = 1000) [3]	BRKGA [11]	PGA [35]	IGA
EX11	**70**	**70**	**70**	**70**	**70**
EX21	-	**74**	76	**74**	**74**
EX41	-	**72**	**72**	**72**	**72**
EX51	**59**	**59**	61	**59**	**59**
EX71	-	**81**	**81**	82	**81**
EX81	-	94	-	-	91 *
EX91	-	**82**	**82**	**82**	**82**
EX12	**56**	**56**	59	**56**	**56**
EX22	**61**	**62**	**62**	**62**	**62**
EX42	-	**56**	58	59	**56**
EX52	**47**	48	49	**47**	**47**
EX72	-	62	62	63	61 *
EX82	-	82	-	-	80 *
EX92	**69**	**69**	**69**	**69**	**69**
EX13	**62**	**62**	**62**	**62**	**62**
EX23	-	**67**	**67**	**67**	**67**
EX43	-	**61**	63	62	**61**
EX53	**52**	**52**	53	52	**52**
EX73	-	**66**	67	67	**66**
EX83	-	85	-	-	84 *
EX93	-	**73**	74	74	**73**
EX14	**78**	**78**	**78**	**78**	**78**
EX24	-	**84**	87	**84**	**84**
EX44	-	**80**	82	**80**	**80**
EX54	**64**	**64**	68	**64**	**64**
EX74	-	**94**	97	95	**94**
EX84	-	**102**	-	-	**102**
EX94	-	**87**	89	**87**	**87**

**Table 4:** Comparison results for the instances in data set 2 with *t*/*p* ≤ 0.25.

Instances	MILP [3]	LAHC (H = 1000) [3]	BRKGA [11]	PGA [35]	IGA
EX110	**94**	**94**	**94**	**94**	**94**
EX210	**104**	**104**	**104**	106	**104**
EX410	-	**92**	**92**	93	**92**
EX510	**77**	**77**	**77**	**77**	**77**
EX710	-	103	**102**	**102**	**102**
EX810	-	**141**	-	-	**141**
EX910	**118**	**118**	119	**118**	**118**
EX120	**91**	**91**	**91**	**91**	**91**
EX220	**102**	**102**	**102**	103	**102**
EX420	**88**	**88**	90	**88**	**88**
EX520	**76**	**76**	**76**	**76**	**76**
EX720	-	99	98	99	**98**
EX820	-	**138**	-	-	**138**
EX920	**116**	116	118	116	**116**
EX130	**92**	**92**	95	**92**	**92**
EX230	**102**	**102**	**102**	**102**	**102**
EX430	**89**	**89**	90	**89**	**89**
EX530	**77**	**77**	78	**77**	**77**
EX730	-	101	100	102	99 *
EX830	-	139	-	-	**139**
EX930	**117**	**118**	**118**	**118**	**118**
EX140	**97**	**97**	99	99	**97**
EX241	**153**	154	**153**	154	**153**
EX441	**131**	134	133	134	**131**
EX541	**113**	**113**	**113**	**113**	**113**
EX740	-	105	**104**	**104**	**104**
EX741	-	150	150	151	149 *
EX840	-	144	-	-	143 *
EX940	**119**	121	121	120	**119**

**Table 5:** Comparison results for the instances in data set 3.

Instances	MILP [3]	LAHC (H = 1000) [3]	PGA [35]	IGA
MFJST01	**485**	**485**	**485**	**485**
MFJST02	**463**	**463**	**463**	**463**
MFJST03	**482**	**482**	**482**	**482**
MFJST04	**576**	**576**	584	**576**
MFJST05	**532**	**532**	542	**532**
MFJST06	**652**	**652**	**652**	**652**
MFJST07	-	**898**	1016	**898**
MFJST08	-	**900**	1214	**900**
MFJST09	-	1120	1415	1117 *
MFJST10	-	1238	1613	1228 *

**Table 6:** Comparison results for the instances in data set 4.

Instances	LAHC (H = 1000) [3]	LAHC (H = 100) [3]	IGA
MKT01	187	197	177 *
MKT02	148	157	126 *
MKT03	371	380	342 *
MKT04	**225**	240	244-
MKT05	312	329	295 *
MKT06	389.5	416.5	321.5 *
MKT07	291	306	267 *
MKT08	846	858	780.5 *
MKT09	794	829.5	715 *
MKT10	712.5	743	645 *

**Table 7:** Comparison results for the instances in data set 5.

Instances	LAHC (H = 1000) [3]	LAHC (H = 100) [3]	IGA
mt10c1t	992	1026	991 *
mt10cct	**973**	982	**973**
mt10xt	991	1003	989 *
mt10xxt	**991**	993	**991**
mt10xxxt	983	1040	981 *
mt10xyt	983	1006	980 *
mt10xyzt	**934**	972	**934**
setb4c9t	1005	1036	1004 *
setb4cct	**979**	1036	990-
setb4xt	**994**	1033	995-
setb4xxt	1018	1045	1017 *
setb4xxxt	993	1019	992 *
setb4xyt	969	1005	963 *
setb4xyzt	979	1041	978 *
seti5c12t	1361	1415	1347 *
seti5cct	1358	1401	1335 *
seti5xt	1365	1430	1355 *
seti5xxt	1409	1418	1373 *
seti5xxxt	1390	1416	1350 *
seti5xyt	1379	1388	1376 *
seti5xyzt	1347	1399	1309 *

## Data Availability

The data that support the findings of this study are available from the corresponding author, upon reasonable request.

## Appendix A

The improved current best solutions for data sets 2 and 3 are given as follows:

EX81:

operation sequence: 2 5 6 1 5 6 2 4 3 4 1 5 2 3 6 1 4 5 6 3
machine selection: 1 4 4 2 2 1 1 3 3 2 2 2 3 3 3 4 4 4 1 1
AGV selection: 2 1 2 1 1 1 1 2 1 1 1 2 2 2 1 2 2 2 1 1

EX72:

operation sequence: 2 6 5 8 7 2 8 6 5 3 4 6 7 1 3 4 8 7 1
machine selection: 1 3 4 4 4 4 2 2 1 4 3 3 3 1 1 1 2 2 3
AGV selection: 2 1 2 1 2 1 1 1 2 1 2 1 1 1 2 2 2 2 2

EX82:

operation sequence: 6 1 5 1 2 3 6 5 6 4 1 3 2 3 5 4 2 4 6 5
machine selection: 2 2 3 3 4 4 1 3 3 2 2 2 1 4 1 1 4 4 1 4
AGV selection: 2 1 2 1 1 2 1 2 2 1 1 2 1 2 1 2 2 2 2 1

EX83:

operation sequence: 1 3 6 1 3 4 2 5 6 5 2 4 5 1 6 6 4 3 5 2
machine selection: 2 2 1 2 4 4 1 1 3 3 3 3 1 2 2 2 4 4 1 1
AGV selection: 2 1 2 1 1 1 2 1 1 2 2 1 1 1 2 1 1 1 1 1

EX730:

operation sequence: 5 6 2 8 1 3 4 5 7 7 3 4 8 2 8 6 1 7 6
machine selection: 1 2 4 1 4 1 2 2 1 3 3 4 4 1 1 2 2 3 3
AGV selection: 2 1 2 1 2 1 2 1 2 1 2 1 1 1 1 1 1 2 1

EX741:

operation sequence: 2 6 7 8 6 3 8 5 7 8 2 1 5 4 7 1 4 3 6
machine selection: 4 2 4 1 4 1 2 2 1 4 3 3 4 1 2 1 2 3 3
AGV selection: 2 1 1 2 1 1 2 1 1 1 1 2 2 1 1 2 1 1 2

EX840:

operation sequence: 5 2 4 6 3 5 1 3 6 4 6 1 2 5 2 3 1 5 4 6
machine selection: 2 2 4 2 4 1 1 1 3 3 3 3 1 3 2 2 4 4 1 4
AGV selection: 2 1 2 1 2 2 1 2 2 2 2 1 1 1 2 1 1 1 1 2

MFJST09:

operation sequence: 7 8 3 7 3 5 10 4 2 5 6 9 4 6 8 7 5 1 3 2 9 11 5 2 7 4 8 3 10 11 6 8 1 9 9 10 1 4 11 1 6 10 11 2
machine selection: 1 2 4 8 1 2 7 5 2 7 5 8 2 5 4 6 1 5 2 8 2 3 5 4 3 3 7 5 4 6 7 7 3 3 5 8 4 6 5 7 2 3 5 6
AGV selection: 2 1 2 1 2 1 1 1 1 2 1 1 1 2 1 1 1 1 1 2 1 1 2 2 1 1 2 1 2 2 1 1 1 2 2 1 1 1 2 1 1 2 2 1

MFJST10:

operation sequence: 5 7 8 3 7 5 11 1 4 10 5 3 12 4 8 3 5 6 4 1 9 2 10 11 7 3 8 12 8 11 1 4 11 2 7 10 1 6 12 2 9 9 6 10 12 6 9 2
machine selection: 1 7 4 8 1 2 6 5 2 7 5 8 2 5 4 6 1 5 6 8 2 4 5 4 3 3 5 5 4 6 7 7 2 3 7 6 4 6 7 7 3 3 5 8 2 3 5 8
AGV selection: 2 1 2 1 2 2 1 1 1 1 2 1 1 1 2 1 2 1 2 2 1 1 2 1 1 1 1 1 2 2 1 2 1 1 1 2 2 1 2 2 1 2 1 1 1 2 1 1

MKT01:

operation sequence: 9 3 1 7 10 5 6 10 5 5 4 1 2 3 3 4 9 8 9 8 10 1 6 6 6 6 7 5 7 7 2 1 10 8 6 8 9 9 4 3 4 8 9 1 5 4 10 2 2 2 10 5 1 3 7
machine selection: 3 3 3 2 3 3 2 3 1 2 2 2 6 6 6 1 2 2 3 3 3 2 2 2 3 4 6 6 1 2 2 2 4 6 1 2 2 3 6 6 2 2 2 6 1 6 1 2 2 3 3 3 6 4 4
AGV selection: 2 1 2 1 1 2 2 1 1 1 1 1 2 1 1 1 1 2 1 1 1 1 1 1 2 2 2 1 2 1 2 1 2 2 1 1 1 1 2 2 2 2 2 1 2 2 2 1 1 2 2 2 1 2 1

MKT02:

operation sequence: 1 7 2 8 1 6 2 10 7 10 2 1 6 8 9 8 3 6 10 3 3 7 4 4 4 4 4 5 9 6 1 10 8 4 5 10 5 7 9 3 2 8 7 2 3 6 9 5 1 6 10 5 1 2 8 9 3 5
machine selection: 6 6 6 2 5 5 5 5 5 2 2 2 1 2 2 5 5 5 2 2 2 2 2 2 4 4 4 4 4 3 6 6 6 6 2 2 5 5 5 5 5 2 2 6 6 6 6 2 6 6 6 2 4 4 4 6 6 6
AGV selection: 2 1 2 1 1 2 1 1 2 2 2 2 2 2 2 2 1 2 2 1 2 2 2 1 1 1 1 1 2 2 2 1 2 2 2 2 2 2 2 2 2 1 1 1 1 1 2 2 2 1 1 1 1 1 1 2 1 1

MKT03:

operation sequence: 9 13 8 10 4 11 15 9 4 1 5 14 1 5 15 8 10 6 7 3 4 7 9 8 2 12 6 14 4 8 2 3 7 15 12 1 9 14 13 3 13 5 9 6 9 3 7 11 11 10 13 8 15 10 2 2 12 5 14 6 15 10 12 3 3 2 1 11 13 12 10 13 5 5 4 10 1 8 9 2 14 3 15 12 1 11 4 1 13 6 3 10 7 15 9 7 10 5 6 11 13 12 4 14 12 1 2 4 6 8 7 15 3 8 14 6 5 5 8 12 9 4 13 11 7 2 9 11 1 14 13 2 7 11 10 6 15 14 6 11 14 4 5 2 15 8 7 12 1 3
machine selection: 4 4 7 5 5 5 5 1 8 8 7 1 2 2 2 7 7 7 8 7 3 3 3 6 6 6 4 4 1 2 3 3 1 1 7 7 5 7 1 5 7 7 5 5 2 2 5 5 5 5 4 7 3 3 3 8 8 8 5 5 1 1 4 1 7 7 5 4 4 6 7 1 4 7 1 1 4 4 4 3 1 1 6 6 6 6 5 2 2 8 1 7 4 4 4 4 5 8 8 8 4 7 7 4 5 1 5 5 5 3 8 8 8 1 1 8 5 5 4 4 5 5 4 7 7 7 4 4 5 8 7 4 2 5 5 4 7 5 1 1 2 6 6 7 7 1 1 4 4 7
AGV selection: 2 1 2 1 1 2 1 2 2 2 1 2 1 1 1 2 2 1 2 1 1 2 2 1 2 1 2 2 2 2 2 2 2 1 1 2 1 1 2 2 1 2 2 2 1 2 1 1 1 1 1 1 2 1 1 1 1 1 1 1 1 1 1 2 2 2 2 2 1 1 1 1 1 2 2 2 1 1 1 1 2 2 1 1 1 2 1 1 1 1 1 2 1 2 2 1 1 2 1 2 2 1 2 1 1 2 1 1 1 2 1 2 2 1 2 1 1 1 1 1 2 2 1 2 1 2 2 1 1 2 1 1 2 2 2 2 2 2 1 1 2 2 2 2 2 1 2 1 1 1

MKT05:

operation sequence: 6 9 6 8 12 4 15 10 8 4 11 13 8 10 5 3 13 2 11 15 11 4 4 9 3 12 8 10 6 5 12 15 6 2 9 13 1 14 7 11 14 5 3 10 3 12 13 2 6 3 9 6 1 4 2 7 14 4 11 3 14 8 5 14 7 10 15 7 9 4 13 9 3 12 1 15 7 10 8 13 12 9 8 15 10 14 6 11 1 15 9 10 5 3 14 5 2 11 10 6 1 1 6 13 8 9
machine selection: 2 1 1 3 4 4 3 2 4 4 2 3 3 1 1 1 4 2 3 4 4 4 4 2 2 4 3 4 4 3 2 2 4 4 3 3 4 4 1 1 1 3 3 2 2 3 3 3 3 4 3 4 4 2 3 4 4 2 4 4 1 1 3 1 1 1 3 2 3 3 4 4 1 1 1 3 4 3 3 4 3 3 2 3 4 4 4 4 4 2 2 1 1 1 1 1 1 4 4 3 4 3 3 2 2 2
AGV selection: 2 1 1 2 1 2 1 2 1 1 1 2 1 1 1 2 1 1 2 2 1 1 1 1 2 2 2 1 1 1 2 2 1 2 1 1 1 2 1 2 1 1 1 1 2 2 2 2 1 2 2 1 2 1 2 2 2 2 1 2 2 2 1 2 2 1 2 2 2 1 2 1 1 2 1 2 1 2 1 1 2 1 2 2 2 1 2 1 2 1 1 1 2 2 1 1 1 2 2 2 2 1 1 1 2 1
